# Correction: Nitric oxide acts as a cotransmitter in a subset of dopaminergic neurons to diversify memory dynamics

**DOI:** 10.7554/eLife.64094

**Published:** 2020-10-22

**Authors:** Yoshinori Asou, Robert P Ray, Xi Long, Daniel Bushey, Karol Cichewicz, Teri-TB Ngo, Brandi Sharp, Christina Christoforou, Amy Hu, Andrew L Lemire, Paul Tillberg, Jay Hirsh, Ashok Litwin-Kumar, Gerald M Rubin

Aso Y, Ray RP, Long X, Bushey D, Cichewicz K, Ngo T-TB, Sharp B, Christoforou C, Hu A, Lemire AL, Tillberg P, Hirsh J, Litwin-Kumar A, Rubin GM. 2019. Nitric oxide acts as a cotransmitter in a subset of dopaminergic neurons to diversify memory dynamics. *eLife* **8**:e49257. doi: 10.7554/eLife.49257.

Published 17, November 2019

We improved our RNA-seq analysis pipeline using the STARsolo aligner (https://github.com/alexdobin/STAR/blob/master/docs/STARsolo.md) in conjunction with a new release of the genome annotation from Flybase (r6.34 instead of the r6.17 used in the original publication) to generate more accurate transcript counts from our sequencing reads. The changes to our published data are minor and all the statistical conclusions are consistent between the original and revised datasets. The changes do not alter any of our conclusions or require revision of the published figures. Nonetheless, we would like to update the Supplementary file 1 to provide the most accurate data for future use. The analysed data was updated at NCBI Gene Expression Ombnibus. The accession number GSE139889 remains the same as in the original publication (https://www.ncbi.nlm.nih.gov/geo/query/acc.cgi?acc=GSE139889).

The original and corrected text in the Materials and methods subsection “Library preparation and sequencing” are shown below with new text indicated in bold.

Original:

Sequencing adapters were trimmed from the reads with Cutadapt (Martin, 2011) prior to alignment with STAR (Dobin et al., 2013) to the *Drosophila* r6.17 genome assembly on Flybase (Thurmond et al., 2019).

Corrected:

Sequencing adapters were trimmed from the reads with Cutadapt (Martin, 2011) prior to alignment with the STARsolo aligner (https://github.com/alexdobin/STAR/blob/master/docs/STARsolo.md) (Dobin et al., 2013; Dobin, 2020) to the *Drosophila* r6.34 genome assembly on Flybase (Thurmond et al., 2019).

The following reference was added:

Dobin A. 2020. STARsolo: mapping, demultiplexing and quantification for single cell RNA-seq. *GitHub*. 2.7.5a. https://github.com/alexdobin/STAR

The article has been corrected accordingly.

