# Supplementary material for: Nitric oxide acts as a cotransmitter in a subset of dopaminergic neurons to diversify memory dynamics
Source: eLife. 2019 Nov 14;8:e49257. doi: 10.7554/eLife.49257 (PMC6948953; doi:10.7554/eLife.49257)
Supplement: Supplementary file 2. — The list of key reagents used in this study. [file elife-49257-supp2.docx]

| **Key Resources Table** | | | | |
| --- | --- | --- | --- | --- |
| **Reagent type (species) or resource** | **Designation** | **Source or reference** | **Identifiers** | **Additional information** |
| gene (*Drosophila melanogaster*) | NOS |  | FLYB:FBgn 0011676 |  |
| gene (*Drosophila melanogaster*) | Gycbeta100B |  | FLYB:FBgn 0013973 |  |
| strain, strain background (E. coli) | T7 Express lysY/lq | New England BioLabs | C3013 |  |
| genetic reagent (*D. melanogaster*) | *20xUAS-CsChrimson-mVenus attP18* | Klapoetke et al., 2014  PMID: 24509633 | N.A. |  |
| genetic reagent (*D. melanogaster*) | *ple2, DTHFS^+/−^ BAC in attP2* | Cichewicz et al., 2017  PMID: 27762066 | N.A. |  |
| genetic reagent (*D. melanogaster*) | *UAS-DTH* | Cichewicz et al., 2017  PMID: 27762066 | N.A. |  |
| genetic reagent (*D. melanogaster*) | *TH-ZpGAL4DBD in VK00027* | Aso et al., 2014a  PMID: 25535793 | N.A. |  |
| genetic reagent (*D. melanogaster*) | *DDC-ZpGAL4DBD in VK00027* | Aso et al., 2014a  PMID: 25535793 | N.A. |  |
| genetic reagent (*D. melanogaster*) | *R52H03-p65ADZp attP40* | Aso et al., 2014a  PMID: 25535793 | N.A. |  |
| genetic reagent (*D. melanogaster*) | *R58E02-p65ADZp attP40* | Aso et al., 2014a  PMID: 25535793 | N.A. |  |
| genetic reagent (*D. melanogaster*) | *Gr66a-LexA* | Aso et al., 2014a  PMID: 25535793 | N.A. |  |
| genetic reagent (*D. melanogaster*) | *13xLexAop2-CsChrimson-mVenus attP18* | Klapoetke et al., 2014  PMID: 24509633 | N.A. |  |
| genetic reagent (*D. melanogaster*) | *pBPp65ADZpUw attP40* | Seeds et al., 2014  PMID: 25139955 | N.A. |  |
| genetic reagent (*D. melanogaster*) | *VT045661-LexAp65 in JK22C* | Aso et al., 2016  PMID: 27441388 | N.A. |  |
| genetic reagent (*D. melanogaster*) | *R73F07-p65ADZp attP40* | Aso et al., 2014a  PMID: 25535793 | N.A. |  |
| genetic reagent (*D. melanogaster*) | *R72B05-p65ADZp attP40* | Aso et al., 2014a  PMID: 25535793 | N.A. |  |
| genetic reagent (*D. melanogaster*) | *R24E12-p65ADZp attP40* | Aso et al., 2014a  PMID: 25535793 | N.A. |  |
| genetic reagent (*D. melanogaster*) | *R58E02-LexAp65 attP40* | Aso et al., 2014a  PMID: 25535793 | N.A. |  |
| genetic reagent (*D. melanogaster*) | *R30E11-p65ADZp attP40* | Aso et al., 2014a  PMID: 25535793 | N.A. |  |
| genetic reagent (*D. melanogaster*) | *R22B12-ZpGDBD JK73A* | this study; |  | Available from Aso lab |
| genetic reagent (*D. melanogaster*) | *UAS-NOS-shRNA strain#1*  y[1] sc[*] v[1]; P{y[+t7.7] v[+t1.8]=TRiP.HMC03076}attP2  (VALIUM20 vector; TCGGAGCAATATGCGAAGCAA) | Bloomington Drosophila Stock Center | 50675 |  |
| genetic reagent (*D. melanogaster*) | *UAS-NOS-shRNA strain#2*  SH09526.N in attP40  (VALIUM20 vector; ACCACTGGACATTATCAGCTA) | this study;  Parkins et al., 2015  PMID: 26320097 |  | Available from Aso lab |
| genetic reagent (*D. melanogaster*) | *RNAi background for attP2* | Bloomington Drosophila Stock Center | 36303 |  |
| genetic reagent (*D. melanogaster*) | *RNAi background for attP40* | Bloomington Drosophila Stock Center | 36304 |  |
| genetic reagent (*D. melanogaster*) | *UAS-NOS* | Bloomington Drosophila Stock Center | 56823 |  |
| genetic reagent (*D. melanogaster*) | *MB-Switch* | Mao et al., 2004  PMID: 14684832 | N.A. |  |
| genetic reagent (*D. melanogaster*) | Gycbeta100B-RNAi  w; P{y[+t7.7] v[+t1.8]=TRiP.HMJ22589}attP40 | Bloomington Drosophila Stock Center | N.A. |  |
| genetic reagent (*D. melanogaster*) | Gycbeta100B-RNAi  *KK100706* | Vienna Drosophila Resource Center  Dietzl et al., 2007  PMID: 17625558 | 100706 |  |
| genetic reagent (*D. melanogaster*) | scrib-RNAi  y[1]sc[*]v[1];P{y[+t7.7]v[+t1.8]=TRiP.HMS01993}attP40/CyO | Bloomington Drosophila Stock Center | 39073 |  |
| genetic reagent (*D. melanogaster*) | Gycbeta100B[MI08892-GFSTF.2] | Bloomington Drosophila Stock Center  Nagarkar-Jaiswal et al., 2015, PMID: 25824290 | 60565 |  |
| genetic reagent (*D. melanogaster*) | *MB320C-split-GAL4* | Aso et al., 2014a  PMID: 25535793 | N.A. |  |
| genetic reagent (*D. melanogaster*) | *MB022B-split-GAL4* | Aso et al., 2014a  PMID: 25535793 | N.A. |  |
| genetic reagent (*D. melanogaster*) | *MB630B-split-GAL4* | Aso et al., 2016  PMID: 27441388 | N.A. |  |
| genetic reagent (*D. melanogaster*) | *13XLexAop2-CsChrimson-tdTomato in attP18* | Klapoetke et al., 2014  PMID: 24509633 | N.A. |  |
| genetic reagent (*D. melanogaster*) | *10xUAS-Syn21-Chrimson88-tdT-3.1 in attP18* | Klapoetke et al., 2014  PMID: 24509633 | N.A. |  |
| genetic reagent (*D. melanogaster*) | *20X-IVS-Syn21-OpGCamp6f-p10 in VK00005* | Chen et al., 2013  PMID: 23868258 | N.A. |  |
| genetic reagent (*D. melanogaster*) | *LexAop2-Syn21-opGCaMP6s in su(Hw)attP8* | Chen et al., 2013  PMID: 23868258 | N.A. |  |
| genetic reagent (*D. melanogaster*) | *20XUAS-IVS-Syn21-opGCaMP6f-p10 in su(Hw)attP8* | Chen et al., 2013  PMID: 23868258 | N.A. |  |
| genetic reagent (*D. melanogaster*) | *VT038111-GAL4 in JK22C* | this study |  | Available from Aso lab |
| genetic reagent (*D. melanogaster*) | UAS-7xHalo7::CAAX in attP40 | Bloomington Drosophila Stock Center  Sutcliffe et al., 2017  PMID: 28209589 | 67621 |  |
| antibody | anti-Brp (Mouse monoclonal) | *Developmental Studies Hybridoma Bank* | nc82; [AB_2314866](http://antibodyregistry.org/AB_2314866) | IF(1:30) |
| antibody | anti-TH (Mouse monoclonal) | Millipore-Sigma | MAB318-AF555; clone LNC1 | IF(1:400) |
| antibody | anti-NOS exon 16 (Rabbit polyclonal) | Yakubovich et al., 2010  PMID: 20178753  This paper | N.A. | IF(1:1000)  preabsorbed with 57C10-GAL4>NOS-RNAi brains |
| antibody | anti-GFP (Chicken polyclonal) | abcam | ab13970 | IF(1:400) |
| antibody | Goat anti-Rabbit IgG (H+L) Highly Cross-Adsorbed Secondary Antibody, Alexa Fluor 568 | Invitrogen | A-11036 | IF(1:200) |
| antibody | Goat anti-Chicken IgY (H+L) Secondary Antibody, Alexa Fluor 488 | Invitrogen | A-11039 | IF(1:200) |
| antibody | Anti-Mouse IgG (H&L) (Goat), ATTO 647N conjugated | ROCKLAND  antibodies & assays | 610-156-121 | IF(1:100) |
| recombinant DNA reagent | pET28a-dNOS exon 16 | Yakubovich et al., 2010  PMID: 20178753 | N.A. |  |
| recombinant DNA reagent | pRSET-dNOS exon 16 | This paper | N.A. |  |
| sequence-based reagent | NOS FISH prob#01_CGCGCCGCAAGCTCAAGA | Biosearch Technologies | N.A. |  |
| sequence-based reagent | NOS FISH prob#02_CCTAGCGCGACTTTTGAC | Biosearch Technologies | N.A. |  |
| sequence-based reagent | NOS FISH prob#03_TCCTGGTGTGTCTCTATT | Biosearch Technologies | N.A. |  |
| sequence-based reagent | NOS FISH prob#04_AACAGTCACTTCACTGGC | Biosearch Technologies | N.A. |  |
| sequence-based reagent | NOS FISH prob#05_TCTTCTCACCTCTCTTTA | Biosearch Technologies | N.A. |  |
| sequence-based reagent | NOS FISH prob#06_ACTTCGTTAACTCACGCC | Biosearch Technologies | N.A. |  |
| sequence-based reagent | NOS FISH prob#07_CTCGGCGTCTTTCAAACG | Biosearch Technologies | N.A. |  |
| sequence-based reagent | NOS FISH prob#08_ACCTCCCATTTAACAGGT | Biosearch Technologies | N.A. |  |
| sequence-based reagent | NOS FISH prob#09_ACCAAGTGGACAACCGAC | Biosearch Technologies | N.A. |  |
| sequence-based reagent | NOS FISH prob#10_TTGCTCCGTTTGTTAGTC | Biosearch Technologies | N.A. |  |
| sequence-based reagent | NOS FISH prob#11_CTCGACGAGGTGCAAGGG | Biosearch Technologies | N.A. |  |
| sequence-based reagent | NOS FISH prob#12_CTGTTCTACAGCGTCGTA | Biosearch Technologies | N.A. |  |
| sequence-based reagent | NOS FISH prob#13_TTGGACGCTAAGCACTGG | Biosearch Technologies | N.A. |  |
| sequence-based reagent | NOS FISH prob#14_CGTGTCTGTGTCGTTGTT | Biosearch Technologies | N.A. |  |
| sequence-based reagent | NOS FISH prob#15_GTTTTGAGTTCGTTGCGG | Biosearch Technologies | N.A. |  |
| sequence-based reagent | NOS FISH prob#16_CAACTTACCGTTACCCGA | Biosearch Technologies | N.A. |  |
| sequence-based reagent | NOS FISH prob#17_ACTCGCCGTTAGGTTTAC | Biosearch Technologies | N.A. |  |
| sequence-based reagent | NOS FISH prob#18_GCCACTGAGGAGCGGGTC | Biosearch Technologies | N.A. |  |
| sequence-based reagent | NOS FISH prob#19_TACTTCACCTGGTAGGCC | Biosearch Technologies | N.A. |  |
| sequence-based reagent | NOS FISH prob#20_GTGTTCCTCGAGTTCGTC | Biosearch Technologies | N.A. |  |
| sequence-based reagent | NOS FISH prob#21_CCTCCGAACGGTAGAAGT | Biosearch Technologies | N.A. |  |
| sequence-based reagent | NOS FISH prob#22_CGGTAACTCCGTGGTGTT | Biosearch Technologies | N.A. |  |
| sequence-based reagent | NOS FISH prob#23_CGCGGTCATAGAGGTGTC | Biosearch Technologies | N.A. |  |
| sequence-based reagent | NOS FISH prob#24_AGCGGAGGTTAGGCGCTT | Biosearch Technologies | N.A. |  |
| sequence-based reagent | NOS FISH prob#25_CGTGGTTGTGGTCGTAGC | Biosearch Technologies | N.A. |  |
| sequence-based reagent | NOS FISH prob#26_CAGCTTGACCTACCGTCA | Biosearch Technologies | N.A. |  |
| sequence-based reagent | NOS FISH prob#27_ACCGCAACCGCAACCAGT | Biosearch Technologies | N.A. |  |
| sequence-based reagent | NOS FISH prob#28_CACGCCCAACAGGAGGGA | Biosearch Technologies | N.A. |  |
| sequence-based reagent | NOS FISH prob#29_GACGTGACGCAGGCCTTT | Biosearch Technologies | N.A. |  |
| sequence-based reagent | NOS FISH prob#30_CAAGCGCCCTTGATAGCG | Biosearch Technologies | N.A. |  |
| sequence-based reagent | NOS FISH prob#31_AGCGGCTTTTTGGTTGTC | Biosearch Technologies | N.A. |  |
| sequence-based reagent | NOS FISH prob#32_CGGGTCTTTCTACAGTGT | Biosearch Technologies | N.A. |  |
| sequence-based reagent | NOS FISH prob#33_TAATAGCCAGCGCACGGC | Biosearch Technologies | N.A. |  |
| sequence-based reagent | NOS FISH prob#34_AATACGTGGACCTGCTGC | Biosearch Technologies | N.A. |  |
| sequence-based reagent | NOS FISH prob#35_GTCAGACGACTACGCGTT | Biosearch Technologies | N.A. |  |
| sequence-based reagent | NOS FISH prob#36_CTGACTTCTTGTAGCTCC | Biosearch Technologies | N.A. |  |
| sequence-based reagent | NOS FISH prob#37_CTCCAGATGCTGTGCGAC | Biosearch Technologies | N.A. |  |
| sequence-based reagent | NOS FISH prob#38_GACGTTCCCAGCGCTCTA | Biosearch Technologies | N.A. |  |
| sequence-based reagent | NOS FISH prob#39_AAAGGACGAGCTTCCGGT | Biosearch Technologies | N.A. |  |
| sequence-based reagent | NOS FISH prob#40_ACATGCTCGTCGTAATAC | Biosearch Technologies | N.A. |  |
| commercial assay or kit | PicoPure RNA Isolation Kit | Life Technologies | NEG772014MC |  |
| commercial assay or kit | AminoLink Immobilization Kit | Thermo Scientific | #44890 |  |
| chemical compound, drug | S-(−)-Carbidopa | Millipore-Sigma | C1335 |  |
| chemical compound, drug | L-DOPA | Millipore-Sigma | D9628 |  |
| chemical compound, drug | L-NNA (Nu-nitro-L-arginin) | Millipore-Sigma | N5501 |  |
| chemical compound, drug | Schneider's *Drosophila* Medium | Thermo Fisher Scientific | 21720024 |  |
| chemical compound, drug | RU-486 | Millipore-Sigma | 475838 |  |
| chemical compound, drug | Cy®5 Mono | GE Healthcare | PA25001 |  |
| chemical compound, drug | Cy®3 Mono | GE Healthcare | PA23001 |  |
| chemical compound, drug | RNase-free water: Molecular Biology Grade Water | Corning | 46-000-CM |  |
| chemical compound, drug | RNase-free 1x PBS | Fisher | BP2438-4 |  |
| chemical compound, drug | CH3COOH: Acetic Acid | Fisher | A38S-500 |  |
| chemical compound, drug | NaBH4: Sodium borohydride, 99%, VenPure™ SF powder | Acros Organics | AC448481000 |  |
| chemical compound, drug | 20xSSC | Fisher | AM9763 |  |
| chemical compound, drug | Hi-Di formamide | Applied Biosystems | 4440753 |  |
| chemical compound, drug | 50x Denhardt's solution | Alfa Aesar | AAJ63135AD |  |
| chemical compound, drug | tRNA: baker's yeast | Roche | 10109495001 |  |
| chemical compound, drug | UltraPure™ Salmon Sperm DNA Solution | Thermofisher | 15632011 |  |
| chemical compound, drug | 10% SDS | Corning | 46-040-CI |  |
| chemical compound, drug | Formamide (Deionized) | Ambion | AM9342 |  |
| chemical compound, drug | Liberase DH | Roche | 5401054001 |  |
| software, algorithm | DE-seq2 | Love et al., 2014  PMID: 25516281 | N.A. |  |
| software, algorithm | Fiji | Schindelin et al., 2012  PMID: 22743772 | N.A. |  |
| software, algorithm | MATLAB | MathWorks | N.A. |  |
| software, algorithm | Prism | GraphPad | N.A. |  |
| software, algorithm | VVD_Viewer/Fluorender | Wang et al., 2012  https://github.com/takashi310/VVD_Viewer/blob/master/README.md | N.A. |  |
