# Supplementary material for: Nitric oxide acts as a cotransmitter in a subset of dopaminergic neurons to diversify memory dynamics
Source: eLife. 2019 Nov 14;8:e49257. doi: 10.7554/eLife.49257 (PMC6948953; doi:10.7554/eLife.49257)
Supplement: Supplementary file 3. — The list of Drosophila genotypes and drug treatment used in each experiment. [file elife-49257-supp3.docx]

**SUPPLEMENTAL File 3**

| **Figure** | **Genotype** | **Drug and other treatments** |
| --- | --- | --- |
| Figure 1D top | *w, 20xUAS-CsChrimson-mVenus in attP18/w; R52H03-p65ADZp attP40/+; TH-ZpGAL4DBD in VK00027/+* |  |
| Figure 1E top | *w, 20xUAS-CsChrimson-mVenus in attP18/w; R52H03-p65ADZp attP40/+; TH-ZpGAL4DBD in VK00027, ple2, DTHFS^+/−^ in attP2/ ple2, DTHFS^+/−^ in attP2* |  |
| Figure 1F top | *w, 20xUAS-CsChrimson-mVenus in attP18/w; R52H03-p65ADZp attP40/+; TH-ZpGAL4DBD in VK00027, ple2, DTHFS^+/−^ in attP2/ ple2, DTHFS^+/−^ in attP2* | 1mg/ml L-Dopa,  0.1 mg/ml S-(−)-  Carbidopa  for 12-16 hours |
| Figure 1G top | *w, 20xUAS-CsChrimson-mVenus in attP18/w; R52H03-p65ADZp attP40/UAS-dTH; TH-ZpGAL4DBD in VK00027, ple2, DTHFS^+/−^ in attP2/ ple2, DTHFS^+/−^ in attP2* |  |
| Figure 1D bottom | *w, 20xUAS-CsChrimson-mVenus in attP18/w; R58E02-p65ADZp attP40/+; DDC-ZpGAL4DBD in VK00027/+* |  |
| Figure 1E bottom | *w, 20xUAS-CsChrimson-mVenus in attP18/w; R58E02-p65ADZp attP40/+; DDC-ZpGAL4DBD in VK00027, ple2, DTHFS^+/−^ in attP2/ ple2, DTHFS^+/−^ in attP2* |  |
| Figure 1F bottom | *w, 20xUAS-CsChrimson-mVenus in attP18/w; R58E02-p65ADZp attP40/+; DDC-ZpGAL4DBD in VK00027, ple2, DTHFS^+/−^ in attP2/ ple2, DTHFS^+/−^ in attP2* | 1mg/ml L-Dopa,  0.1 mg/ml S-(−)-  Carbidopa  for 12-16 hours |
| Figure 1G bottom | *w, 20xUAS-CsChrimson-mVenus in attP18/w; R58E02-p65ADZp attP40/UAS-dTH; DDC-ZpGAL4DBD in VK00027, ple2, DTHFS^+/−^ in attP2/ ple2, DTHFS^+/−^ in attP2* |  |
| Figure 1-figure supplement 1 PAM Wild type | *w, 20xUAS-CsChrimson-mVenus in attP18/w; R58E02-p65ADZp attP40/+; DDC-ZpGAL4DBD in VK00027/+* |  |
| Figure 1- figure supplement 1 PAM Dopamine null | *w, 20xUAS-CsChrimson-mVenus in attP18/w; R58E02-p65ADZp attP40/+; DDC-ZpGAL4DBD in VK00027, ple2, DTHFS^+/−^ in attP2/ ple2, DTHFS^+/−^ in attP2* |  |
| Figure 1- figure supplement 1 PAM Dopamine null TH rescue | *w, 20xUAS-CsChrimson-mVenus in attP18/w; R58E02-p65ADZp attP40/UAS-dTH; DDC-ZpGAL4DBD in VK00027, ple2, DTHFS^+/−^ in attP2/ ple2, DTHFS^+/−^ in attP2* |  |
| Figure 1-figure supplement 1 PPL1 Dopamine null | *w, 20xUAS-CsChrimson-mVenus in attP18/w; R52H03-p65ADZp attP40/+; TH-ZpGAL4DBD in VK00027, ple2, DTHFS^+/−^ in attP2/ ple2, DTHFS^+/−^ in attP2* |  |
| Figure 1- figure supplement 1 PPL1 TH rescue | *w, 20xUAS-CsChrimson-mVenus in attP18/w; R52H03-p65ADZp attP40/UAS-dTH; TH-ZpGAL4DBD in VK00027, ple2, DTHFS^+/−^ in attP2/ ple2, DTHFS^+/−^ in attP2* |  |
| Figure 1- figure supplement 2 | *w, 20xUAS-CsChrimson-mVenus in attP18/w; R52H03-p65ADZp attP40/+; TH-ZpGAL4DBD in VK00027, ple2, DTHFS^+/−^ in attP2/ ple2, DTHFS^+/−^ in attP2* | 1mg/ml L-Dopa,  0.1 mg/ml S-(−)-  Carbidopa |
| Figure 2 | *w, 20xUAS-CsChrimson-mVenus in attP18/w; Gr66a-GAL4/+; +/ ple2, DTHFS^+/−^ in attP2* |  |
| Figure 2 | *w, 20xUAS-CsChrimson-mVenus in attP18/w; Gr66a-GAL4/+; ple2, DTHFS^+/−^ in attP2/ TH-ZpGAL4DBD in VK00027, ple2, DTHFS^+/−^ in attP2* |  |
| Figure 2 | *w, 20xUAS-CsChrimson-mVenus in attP18/w; R52H03-p65ADZp attP40/+; +/TH-ZpGAL4DBD in VK00027, ple2, DTHFS^+/−^ in attP2* |  |
| Figure 2 | *w, 20xUAS-CsChrimson-mVenus in attP18/w; R52H03-p65ADZp attP40/+; ple2, DTHFS^+/−^ in attP2/TH-ZpGAL4DBD in VK00027, ple2, DTHFS^+/−^ in attP2* |  |
| Figure 2 | *w, 13xLeXAop-CsChrimson-tdTomato attp18/w; VT045661-LexA JK22C/+; +/TH-ZpGAL4DBD in VK00027, ple2, DTHFS^+/−^ in attP2* |  |
| Figure 2 | *w, 13xLeXAop-CsChrimson-tdTomato attp18/w; VT045661-LexA JK22C/+; ple2, DTHFS^+/−^ in attP2/ TH-ZpGAL4DBD in VK00027, ple2, DTHFS^+/−^ in attP2* |  |
| Figure 2 | *w, 20xUAS-CsChrimson-mVenus in attP18/w; R73F07-p65ADZp attP40/+; +/TH-ZpGAL4DBD in VK00027, ple2, DTHFS^+/−^ in attP2* |  |
| Figure 2 | *w, 20xUAS-CsChrimson-mVenus in attP18/w; R73F07-p65ADZp attP40/+; ple2, DTHFS^+/−^ in attP2/TH-ZpGAL4DBD in VK00027, ple2, DTHFS^+/−^ in attP2* |  |
| Figure 2 | *w, 20xUAS-CsChrimson-mVenus in attP18/w; R72B05-p65ADZp attP40/+; +/TH-ZpGAL4DBD in VK00027, ple2, DTHFS^+/−^ in attP2* |  |
| Figure 2 | *w, 20xUAS-CsChrimson-mVenus in attP18/w; R72B05-p65ADZp attP40/+; ple2, DTHFS^+/−^ in attP2/TH-ZpGAL4DBD in VK00027, ple2, DTHFS^+/−^ in attP2* |  |
| Figure 2 | *w, 20xUAS-CsChrimson-mVenus in attP18/w; R24E12-p65ADZp attP40/+; +/DDC-ZpGAL4DBD in VK00027, ple2, DTHFS^+/−^ in attP2* |  |
| Figure 2 | *w, 20xUAS-CsChrimson-mVenus in attP18/w; R24E12-p65ADZp attP40/+; ple2, DTHFS^+/−^ in attP2/DDC-ZpGAL4DBD in VK00027, ple2, DTHFS^+/−^ in attP2* |  |
| Figure 2 | *w, 20xUAS-CsChrimson-mVenus in attP18/w; R58E02-p65ADZp attP40/+; TH-ZpGAL4DBD in VK00027/+* |  |
| Figure 2 | *w, 20xUAS-CsChrimson-mVenus in attP18/w; R58E02-p65ADZp attP40/+; ple2, DTHFS^+/−^ in attP2/TH-ZpGAL4DBD in VK00027, ple2, DTHFS^+/−^ in attP2* |  |
| Figure 2 | *w, 20xUAS-CsChrimson-mVenus in attP18/w; R58E02-p65ADZp attP40/+; +/DDC-ZpGAL4DBD in VK00027, ple2, DTHFS^+/−^ in attP2* |  |
| Figure 2 | *w, 20xUAS-CsChrimson-mVenus in attP18/w; R58E02-p65ADZp attP40/+; ple2, DTHFS^+/−^ in attP2/DDC-ZpGAL4DBD in VK00027, ple2, DTHFS^+/−^ in attP2* |  |
| Figure 2- figure supplement 1 left | *w, 20xUAS-CsChrimson-mVenus in attP18/w; R73F07-p65ADZp attP40/+; +/TH-ZpGAL4DBD in VK00027, ple2, DTHFS^+/−^ in attP2* |  |
| Figure 2- figure supplement 1 center | *w, 20xUAS-CsChrimson-mVenus in attP18/w; R72B05-p65ADZp attP40/+; +/TH-ZpGAL4DBD in VK00027, ple2, DTHFS^+/−^ in attP2* |  |
| Figure 2- figure supplement 1 right | *w, 20xUAS-CsChrimson-mVenus in attP18/w; R24E12-p65ADZp attP40/+; +/DDC-ZpGAL4DBD in VK00027, ple2, DTHFS^+/−^ in attP2* |  |
| Figure 3D | *w, 20xUAS-CsChrimson-mVenus in attP18/w;;+/MB320C* |  |
| Figure 3E | *CS* |  |
| Figure 3F left, 3G | *w, 20xUAS-CsChrimson-mVenus in attP18/w;;+/MB320C* |  |
| Figure 3F right | *w, 20xUAS-CsChrimson-mVenus in attP18/w;+;+/MB022B* |  |
| Figure 3-figure supplement 1B | *w, 20xUAS-CsChrimson-mVenus in attP18/w;;* *background attP2 (BDSC#36303)/MB320C* |  |
| Figure 3-figure supplement 1B | *w, 20xUAS-CsChrimson-mVenus in attP18/w;;* *UAS-NOS-shRNA*  *HMC03076 in attP2/MB320C* |  |
| Figure 3-figure supplement 1C | *CS* |  |
| Figure 3- figure supplement 1D | *w/w; UAS-7xHalo7::CAAX in attP40/+;MB320C/+* |  |
| Figure 4A | *w, 13xLeXAop-CsChrimson-tdTomato attp18/w; VT045661-LexA JK22C/+; ple2, DTHFS^+/−^ in attP2/TH-ZpGAL4DBD in VK00027, ple2, DTHFS^+/−^ in attP2* | 0-100 mM L-NNA, 100 mM L-NNA + 1mg/ml L-Dopa,  0.1 mg/ml S-(−)-  Carbidopa, or 1mg/ml L-Dopa,  0.1 mg/ml S-(−)-  Carbidopa for 12-16 hours |
| Figure 4B left | *w, 20xUAS-CsChrimson-mVenus in attP18/w; R58E02-p65ADZp attP40/+; TH-ZpGAL4DBD in VK00027, ple2, DTHFS^+/−^ in attP2/ ple2, DTHFS^+/−^ in attP2* | 0 or 100 mM L-NNA  for 12-16 hours |
| Figure 4B right | *w, 20xUAS-CsChrimson-mVenus in attP18/w; R73F07-p65ADZp attP40/+; TH-ZpGAL4DBD in VK00027, ple2, DTHFS^+/−^ in attP2/ ple2, DTHFS^+/−^ in attP2* | 0 or 100 mM L-NNA  for 12-16 hours |
| Figure 4C left | *w, 20xUAS-CsChrimson-mVenus in attP18/w; R52H03-p65ADZp attP40/background control attP40; TH-ZpGAL4DBD in VK00027, ple2, DTHFS^+/−^ in attP2/ ple2, DTHFS^+/−^ in attP2* |  |
| Figure 4C right | *w, 20xUAS-CsChrimson-mVenus in attP18/w; R52H03-p65ADZp attP40/UAS-NOS-shRNA in attP40; TH-ZpGAL4DBD in VK00027, ple2, DTHFS^+/−^ in attP2/ ple2, DTHFS^+/−^ in attP2* |  |
| Figure 4D and E left | *w, 20xUAS-CsChrimson-mVenus in attP18/w; R72B05-p65ADZp attP40/+; TH-ZpGAL4DBD in VK00027, ple2, DTHFS^+/−^ in attP2/ ple2, DTHFS^+/−^ in attP2* |  |
| Figure 4D and E right | *w, 20xUAS-CsChrimson-mVenus in attP18/w; R72B05-p65ADZp attP40/UAS-NOS; TH-ZpGAL4DBD in VK00027, ple2, DTHFS^+/−^ in attP2/ ple2, DTHFS^+/−^ in attP2* |  |
| Figure 4- figure supplement 1  left | *w, 20xUAS-CsChrimson-mVenus in attP18/w; R73F07-p65ADZp attP40/ +; ple2, DTHFS^+/−^ in attP2/TH-ZpGAL4DBD in VK00027, ple2, DTHFS^+/−^ in attP2* |  |
| Figure 4- figure supplement 1  right | *w, 20xUAS-CsChrimson-mVenus in attP18/w; R73F07-p65ADZp attP40/ UAS-NOS; ple2, DTHFS^+/−^ in attP2/TH-ZpGAL4DBD in VK00027, ple2, DTHFS^+/−^ in attP2* |  |
| Figure 5C left  control (no RNAi) | *w, 13xLeXAop-CsChrimson-tdTomato attp18/w; VT045661-LexA JK22C/background control attp40;MB-switch-GAL4, ple2, DTHFS^+/−^ in attP2 /ple2, DTHFS^+/−^ in attP2* | 0 or 1.5 mg/mL RU486  for 2 days |
| Figure 5C left  Gycbeta100B-RNAi | *w, 13xLeXAop-CsChrimson-tdTomato attp18/w; VT045661-LexA JK22C/UAS-Gycbeta100B-shRNA in attp40;MB-switch-GAL4, ple2, DTHFS^+/−^ in attP2 /ple2, DTHFS^+/−^ in attP2* | 0 or 1.5 mg/mL RU486  for 2 days |
| Figure 5C right  L-Dopa | *w, 13xLeXAop-CsChrimson-tdTomato attp18/w; VT045661-LexA JK22C/UAS-Gycbeta100B-shRNA in attp40;MB-switch-GAL4, ple2, DTHFS^+/−^ in attP2 /ple2, DTHFS^+/−^ in attP2* | 0 or 1.5 mg/mL RU486 and 1mg/ml L-Dopa,  0.1 mg/ml S-(−)-  Carbidopa for 2 days |
| Figure 5D  control (no RNAi) | *w, 13xLeXAop-CsChrimson-tdTomato attp18/w; VT045661-LexA JK22C/UAS-*  *;MB-switch-GAL4, ple2, DTHFS^+/−^ in attP2 /ple2, DTHFS^+/−^ in attP2* |  |
| Figure 5D  scrib-RNAi | *w, 13xLeXAop-CsChrimson-tdTomato attp18/w; VT045661-LexA JK22C/UAS-scrib-shRNA HMS01993 in attp40;MB-switch-GAL4, ple2, DTHFS^+/−^ in attP2 /ple2, DTHFS^+/−^ in attP2* |  |
| Figure 5- figure supplement 1A left | *yw;Gycbeta100B[MI08892-GFSTF.2]* |  |
| Figure 5- figure supplement 1A right | *CS* |  |
| Figure 5- figure supplement 1B left | *yw/w;+/ background control attP40; Gycbeta100B[MI08892-GFSTF.2]/MB-switch-GAL4* | 1.5 mg/mL RU486  for 2 days |
| Figure 5- figure supplement 1B right | *yw/w;+/Gycbeta100B-shRNA in attp40; Gycbeta100B[MI08892-GFSTF.2]/MB-switch-GAL4* | 1.5 mg/mL RU486  for 2 days |
| Figure 5- figure supplement 1C  control (no RNAi) | *yw/w;+/ background control attP40; Gycbeta100B[MI08892-GFSTF.2]/MB-switch-GAL4* | 0 or 1.5 mg/mL RU486  for 2 days |
| Figure 5- figure supplement 1C  Gycbeta100B-RNAi | *yw/w;+/Gycbeta100B-shRNA in attp40; Gycbeta100B[MI08892-GFSTF.2]/MB-switch-GAL4* | 0 or 1.5 mg/mL RU486  for 2 days |
| Figure 5- figure supplement 1D  control (no RNAi) | *w, 13xLeXAop-CsChrimson-tdTomato attp18/w; VT045661-LexA JK22C/background control ;MB-switch-GAL4, ple2, DTHFS^+/−^ in attP2 /ple2, DTHFS^+/−^ in attP2* | 0 or 1.5 mg/mL RU486  for 2 days |
| Figure 5- figure supplement 1D  Gycbeta100B-RNAi | *w, 13xLeXAop-CsChrimson-tdTomato attp18/w; VT045661-LexA JK22C/* Gycbeta100B-RNAi *KK100706;MB-switch-GAL4, ple2, DTHFS^+/−^ in attP2 /ple2, DTHFS^+/−^ in attP2* | 0 or 1.5 mg/mL RU486  for 2 days |
| Figure 5- figure supplement 1E and F | *w/w;+/pJFRC32-10XUAS-IVS-nlsGFP in attP40;+/MB-switch-GAL4* | 0 or 1.5 mg/mL RU486  for 2 days |
| Figure 5- figure supplement 1E and F | *w/w;+/* *pJFRC12-10XUAS-IVS-myr::GFP in attP40;+/MB-switch-GAL4* | 0 or 1.5 mg/mL RU486  for 2 days |
| Figure 5- figure supplement 2A | *w/w, 13XLexAop2-CsChrimson-tdTomato in attP18, 20XUAS-IVS-Syn21-opGCaMP6f-p10 in su(Hw)attP8; +/VT45561-LexA in attp40, VT38111-GAL4 in JK22C ;* *20X-IVS-Syn21-OpGCamp6f-p10 in VK00005 ple2, DTHFS^+/−^ in attP2/ TM3 Sb* | 100mM L-NNA  for 0 or 12-16 hours |
| Figure 5- figure supplement 2A | *w/w, 13XLexAop2-CsChrimson-tdTomato in attP18, 20XUAS-IVS-Syn21-opGCaMP6f-p10 in su(Hw)attP8; +/VT45561-LexA in attp40, VT38111-GAL4 in JK22C ;* *20X-IVS-Syn21-OpGCamp6f-p10 in VK00005 ple2, DTHFS^+/−^ in attP2/ ple2, DTHFS^+/−^ in attP2* | 100mM L-NNA  for 0 or 12-16 hours |
| Figure 5- figure supplement 2B | *w/w, LexAOP2-Syn21-opGCaMP6s in su(Hw)attP8, 10xUAS-Syn21-Chrimson88-tdT-3.1 in attP18; 58E02-LexAp65 attP40/ 30E11-p65ADZp attP40; ple2, DTHFS^+/−^ in attP2/ 22B12-ZpGDBD JK73A, ple2, DTHFS^+/−^ in attP2* |  |
| Figure 5- figure supplement 3B | *w, 20xUAS-CsChrimson-mVenus in attP18/w;;* *background attP2 (BDSC#36303)/MB320C* |  |
| Figure 5- figure supplement 3B | *w, 20xUAS-CsChrimson-mVenus in attP18/w;;* *UAS-NOS-shRNA*  *HMC03076 in attP2/MB320C* |  |
| Figure 5- figure supplement 3C | *w, 13xLeXAop-CsChrimson-tdTomato attp18/w; VT045661-LexA JK22C/+; +/TH-ZpGAL4DBD in VK00027, ple2, DTHFS^+/−^ in attP2* |  |
| Figure 5- figure supplement 3D | *w, 20xUAS-CsChrimson-mVenus in attP18/w; R52H03-p65ADZp attP40/+; TH-ZpGAL4DBD in VK00027, ple2, DTHFS^+/−^ in attP2/ ple2, DTHFS^+/−^ in attP2* |  |
| Figure 5- figure supplement 3D | *w, 20xUAS-CsChrimson-mVenus in attP18/w; R52H03-p65ADZp attP40/UAS-dTH; TH-ZpGAL4DBD in VK00027, ple2, DTHFS^+/−^ in attP2/ ple2, DTHFS^+/−^ in attP2* |  |
| Figure 5- figure supplement 3E | *w, 13xLeXAop-CsChrimson-tdTomato attp18/w; VT045661-LexA JK22C/+; +/TH-ZpGAL4DBD in VK00027, ple2, DTHFS^+/−^ in attP2* |  |
| Figure 6B Wild Type | *w, 13xLeXAop-CsChrimson-tdTomato attp18/w; VT045661-LexA JK22C/+; /+; +/+* |  |
| Figure 6B L-NNA | *w, 13xLeXAop-CsChrimson-tdTomato attp18/w; VT045661-LexA JK22C/+; /+; +/+* | 100mM L-NNA  for 12-16 hours |
| Figure 6B DA null | *w, 13xLeXAop-CsChrimson-tdTomato attp18/w; VT045661-LexA JK22C/+; ple2, DTHFS^+/−^ in attP2/TH-ZpGAL4DBD in VK00027, ple2, DTHFS^+/−^ in attP2* |  |
| Figure 6C | *w, 13xLeXAop-CsChrimson-tdTomato attp18/w; VT045661-LexA JK22C/+; ple2, DTHFS^+/−^ in attP2/TH-ZpGAL4DBD in VK00027, ple2, DTHFS^+/−^ in attP2* |  |
| Figure 6D | *w, 20xUAS-CsChrimson-mVenus in attP18/w;;* *background attP2 (BDSC#36303)/MB320C* |  |
| Figure 6D | *w, 20xUAS-CsChrimson-mVenus in attP18/w;;* *UAS-NOS-shRNA*  *HMC03076 in attP2/MB320C* |  |
| Figure 6E left | *w, 20xUAS-CsChrimson-mVenus in attP18/yw;;* *background attP2 (BDSC#36303)/MB320C* |  |
| Figure 6E center | *w, 20xUAS-CsChrimson-mVenus in attP18/yw;;* *UAS-NOS-shRNA*  *HMC03076 in attP2/MB320C* |  |
| Figure 6E right | *w, 13xLeXAop-CsChrimson-tdTomato attp18/w; VT045661-LexA JK22C/+; ple2, DTHFS^+/−^ in attP2/TH-ZpGAL4DBD in VK00027, ple2, DTHFS^+/−^ in attP2* |  |
| Figure 6-figure supplement 1A | *w, 13xLeXAop-CsChrimson-tdTomato attp18/w; VT045661-LexA JK22C/+; +/+* |  |
| Figure 6-figure supplement 1B control | *w/yw;; background attP2 (BDSC#36303)/MB320C* |  |
| Figure 6- figure supplement 1B NOS-RNAi | *w/yw;;UAS-NOS-shRNA HMC03076 in attP2/MB320C* |  |
| Figure 6- figure supplement 1C | *w, 13xLeXAop-CsChrimson-tdTomato attp18/w; VT045661-LexA JK22C/+; +/+* | 0 or 100mM L-NNA  for 12-16 hours |
| Figure 7B-E Wild Type | *w, 13xLeXAop-CsChrimson-tdTomato attp18/w; VT045661-LexA JK22C/+; /+; +/+* |  |
| Figure 7B-E L-NNA | *w, 13xLeXAop-CsChrimson-tdTomato attp18/w; VT045661-LexA JK22C/+; /+; +/+* | 100mM L-NNA  for 12-16 hours |
| Figure 7B-E DA null | *w, 13xLeXAop-CsChrimson-tdTomato attp18/w; VT045661-LexA JK22C/+; ple2, DTHFS^+/−^ in attP2/TH-ZpGAL4DBD in VK00027, ple2, DTHFS^+/−^ in attP2* |  |
| Figure 9A | *w, 20xUAS-CsChrimson-mVenus in attP18/w;+/MB630B* |  |
